# Scrutinising what Open Access Journals Mean for Global Inequalities

**DOI:** 10.1007/s12109-020-09771-9

**Published:** 2020-11-08

**Authors:** Márton Demeter, Ronina Istratii

**Affiliations:** 1 National University of Public Service, Ludovika Square 2, Annex Building (Szárnyépület), Office 223, 1083 Budapest, Hungary; 2 SOAS University of London, Flat 5 Aylmer Court, Sheldon Avenue, London, N2 0BU UK

**Keywords:** Global publishing, Open access models, Author processing charge, Impact factor, Knowledge production

## Abstract

In the current article, we tested our hypothesis by which high-impact journals tend to have higher Article Processing Charges (APCs) by comparing journal IF metrics with the OA publishing fees they charge. Our study engaged with both journals in Science, Technology, Engineering and Mathematics (STEM) fields and the Humanities and Social Sciences (HSS) and included Hybrid, Diamond and No OA journals. The overall findings demonstrate a positive relationship between APCs and journals with high IF for two of the subject areas we examined but not for the third, which could be mediated by the characteristics and market environment of the publishers. We also found significant differences between the analysed research fields in terms of APC policies, as well as differences in the relationship between APCs and the IF across periodicals. The study and analysis conducted reinforces our concerns that Hybrid OA models are likely to perpetuate inequalities in knowledge production.

## Background and Objectives of Study

In recent years, open access publishing has become a popular concept [6, 12, 19, 20, 22, 28, 31], as evidenced in the latest European initiative by cOAlition S known as Plan S. Increasingly, more and more journals feel compelled to move to an Open Access (OA) business model both for ethical reasons, as have been articulated by the scholarly community [29], and in order to adapt to the ever-shifting publishing market (Open Letter in Support of Funder Open Publishing Mandates; European Commission Statement, 2018). Formerly, Isratii and Demeter problematized the implications of Plan S for global knowledge production and outlined what could be its unintended consequences for knowledge production, focusing on the humanities and social sciences [9, 16].

In that article we made the observation that high-impact journals tend to have higher Article Processing Charges (APCs) and we expressed the concern that a move toward an OA model whereby APCs are covered by the authors and their institutions or funders [12] can reinforce economic and epistemological disparities between researchers in the Global North and South, which we understand in scientometric terms.^1^ The assumption circulating in the various narratives around Plan S seems to be that APCs and journal impact factor (IF) need not be linearly correlated, which would be alarming, but rather that higher APCs could be charged by lower-impact journals, or, conversely that lower APCs could be charged by a high-impact journal [30]. In contrast, we are anticipating that journals with higher IF metrics will tend to charge higher APCs, which will have important implications for knowledge production. Our hypothesis has been that as more and more journals move to a Hybrid Open Access (OA) model (the more typical model encountered currently), relying on APCs to cover their costs and to maintain a profitable existence, if APCs show a tendency to move along with IF, the shift will contribute to maintaining the dominance of publications by Northern researchers in high-impact journals, and consequently, also their hegemony in the realm of scholarly paradigm-setting.

In the current article, we have undertaken to test this hypothesis by comparing journal IF metrics with the OA publishing fees they charge. Our study engaged with both journals in Science, Technology, Engineering and Mathematics (STEM) fields and the Humanities and Social Sciences (HSS) and included Hybrid, Diamond and No OA journals. The overall findings demonstrate a positive relationship between APCs and journals with high IF for two of the subject areas we examined but not for the third, which could be mediated by the characteristics and market environment of the publishers. However, we also found significant differences between the analysed research fields in terms of APC policies, as well as differences in the relationship between APCs and the IF across periodicals. The study and analysis conducted reinforces our concerns that Hybrid OA models are likely to perpetuate inequalities in knowledge production.

This study contributes to a growing literature around the de-Westernization of academic scholarship and the sciences [2, 3, 5, 11, 14]. Extensive research has demonstrated that the global academy is characterised by a significant Global North-Global South imbalance [10]. Editorial boards, selection committees, funding agencies, publishing houses are predominantly located in Northern high-income societies, and the share of Western authors in global knowledge production is overwhelming when contrasted to the contribution of authors from the Global South [21, 24–27]. The current study shows that these inequalities can be further reinforced due to OA publishing models further favouring Northern authors, hindering Southern authors from reaping the benefits of OA publishing models.

## Methods

For this study, we selected to work with the Web of Science (WoS), an established journal indexing database and evaluation platform that covers most academic disciplines. The WoS includes the Journal Citation Report (JCR), which calculates and provides a ranking of journal impact factor (IF) metrics annually. This enabled us to obtain IF values for the journals selected in the current study.

For this exercise, we considered the journals that were indexed to the Social Science Citation index (SSCI) list, which contains journals that have an IF. A more inclusive index, the Emerging Sources Citation Index (ESCI) is also available on the WoS, but the WoS does not calculate IF values for ESCI journals. Subsequently, our analysis considered only the journals indexed on the SSCI list. Since IF values are calculated on the basis of the number of citations that a given journal achieves in a three-year period, by definition, journals with higher IF metrics have greater number of citations and, consequently, greater ‘impact’, in the strict sense that they are more likely to be cited by scholars in their fields and to influence paradigms.^2^ Additionally, journals that wish to be indexed in the SSCI list are assessed by their performance in citations, which means that, by definition, SSCI journals have greater scholarly impact than those journals that are not indexed there.

Our objective was to include journals from both STEM and HSS fields to achieve a more comprehensive study that would consider disciplinary differences. In selecting what fields of study to examine we considered practical and strategic parameters. These needed to have a relatively broad appeal and not be too specialised that could display a very idiosyncratic publishing landscape with limited broader relevance. We also hoped to compare a field of study considered to be highly international with one known to be dominated by Northern publishers to test possible differences in the IF-APCs relationship. We also needed to consider the number of journals under each field of studies selected for analysis to ensure that sampling would be manageable within the timeframe of this study and given the resource constraints that we faced during the COVID-19 pandemic.

**Fig. 1:**
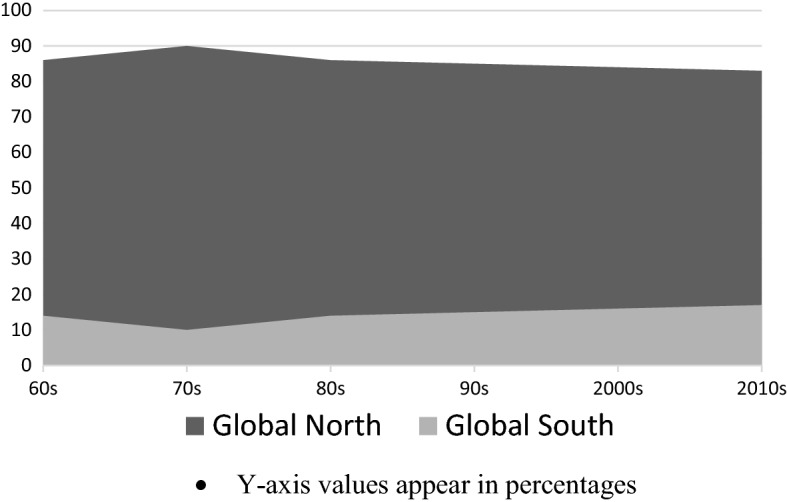
Distribution of development studies publication outputs according to word regions from the 1960 s to the 2010 s. **The Global North/South classification follows the definition given earlier in the article Source: Demeter [10]

Working within these specifications, we initially selected to look at Computer Science and Development Studies. We chose Development Studies because it is historically documented and empirically evidenced that this discipline is extremely biased in favour of the Global North [4, 10, 13, 15, 18]. In 2017, Sarah Cummings and Paul Hoebink co-authored a study that examined patterns of publication in the field of development studies by looking at 10 ‘well-known’ journals [4]. Their findings pointed to a strong Western dominance in terms of the ownership of the journals, the nationality of the authors, and the international diversity of their editorial boards. In his 2020 monograph [10], Márton Demeter demonstrated that all the highly ranked journals in Development Studies were published in Western countries, with the biggest share appertaining to the UK (51%), the US (37%) and the rest of Western Europe (12%). He also found that publication outputs have been historically dominated by the Global North (see Fig. 1).

However, the WoS does list a Development Studies category. As an alternative, we considered using the search engine Scopus, which listed both disciplines. However, Scopus does not use the IF metric, rather the SCImago Journal Rank (SJR). The closest category to Development Studies that we could identify on the WoS was Area Studies and we decided to select this as our second field of study. On the other hand, Computer Science is a fairly international field of studies, which could serve well our purposes. Since there are various subfields within the computer sciences on the WoS, we decided to choose an applied (Computer Science Engineering) and a more theoretical (Computer Science Theoretical) subfield. We anticipated the theoretical field to be dominated by scholarship produced in industrialised societies, and the practical field to display a more even global distribution. This hypothesis was informed by two empirical realities: the unequal distribution of academic capital [10], with western industrialised societies historically dominating in paradigm-setting due to epistemological and material advantages [16], and the observation that practical professions tend to be widespread in industrialising societies appertaining to the Global South. We decided to select Anthropology as a third subject, which has been undoubtedly dominated by Northern researchers. This is also confirmed by the geographic distribution of the journals in the WoS ESCI list, which includes 217 journals in Anthropology, of which 189 (87% of the sample) are published in North America or Western Europe. Moreover, the Euro-American region publishes the top ranking (q1-ranked) journals in the world.

The data we worked with were obtained from the official WoS site. The number of journals in each of the examined fields was as follows: Computer Science Theoretical (*n* = 103), Computer Science Engineering (*n* = 104), Area Studies (*n* = 68), and Anthropology (*n* = 85). We recorded the following data from all the journals: rank on the SSCI list, IF values for 2018, and APCs charged to authors wishing to publish under a Hybrid Open Access (OA) model. We categorized journals according to three OA models: Hybrid OA, Diamond OA and No OA option. In the Hybrid OA model, authors can publish either under the classical model where they do not have to pay APCs but their papers are published behind a paywall (with readers incurring the cost of accessing them) or they can publish open access if they cover the Gold OA APCs. In the latter case, authors (typically with the support of funds granted by their institutions or funders) need to cover the APCs in full, while readers can access the content freely and immediately [12]. In the Diamond OA model, neither the author nor the readers have to pay since Diamond OA papers are free-of-charge for both the authors and the readers. Finally, in journals without an OA publishing possibility, authors must publish under the classical model: they do not need to pay APCs but readers need to pay the journal in order to access the papers [19, 31]. Our initial aim was to include only Gold OA publishing journals, but they are not generally encountered as such, unless they are some kind of so-called ‘predatory’ initiative, charging moderate fees in return for a quicker, less rigorous peer review and editing process.^3^ We did not record data on Green Open Access model, because, in our experience, they offer only a limited contribution to open science. Big publishing houses like Elsevier, Springer and Taylor & Francis demand an embargo period ranging from 18 to 36 months before authors can upload their final manuscripts to public repositories. This can result in a situation whereby papers become out-dated by the time the embargo period is lifted. While all the big publishing houses emphasise the advantages of Gold OA publication model (financed by the author), comprised in a higher number of readers and more citations, they cite no data to demonstrate the same result for delayed Green OA publishing.

Furthermore, we decided to compare APCs with data from different regions of the world in order to explore global economic disparities and the implications for authors in the Global South.^4^ Under Plan S, OA publishing will be encouraged through a strengthening of the link between publication and research funding. As we noted in our previous paper, while OA costs will be covered by funders directly, this is no reassurance from the perspective of global publishing inequalities because of disparities in research funding in the world and the structural and normative characteristics of collaborative research development practices placing researchers outside of Western Europe and North America at a disadvantage [16, 17]. In considering what measures to use for this exercise, we discussed the respective benefits and shortfalls of per capita Gross Domestic Product (GDP) and Purchasing Power Parity (PPP). We decided that it would be important to take into account geographical differences in purchasing power, which would enable us to make a more informed argument about economic disparities among researchers in the world. Finally, in order to be able to relate this exercise to publishing asymmetries and to understand better the implications of the IF-APCs relationship, we analysed the concentration of publishers in high-income societies for the subject areas we examined.

## Results

### APCs- IF Relationship

**Fig. 2:**
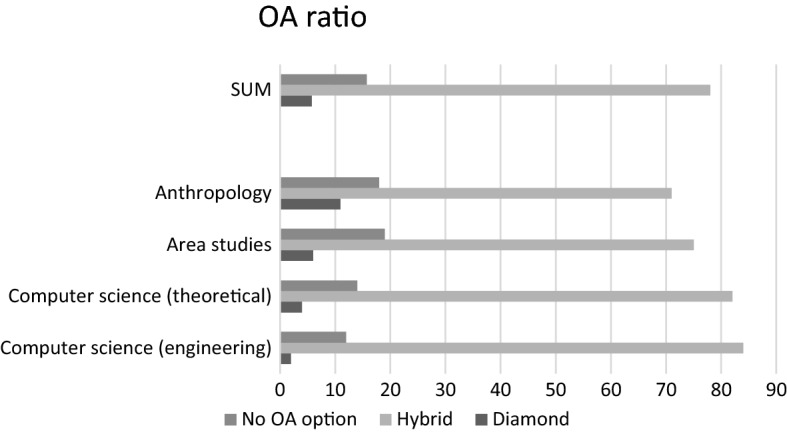
Number of journals by OA model and discipline

Article Processing Charges and OA models were identified for the four subject areas. We then tested correlation using both Pearson correlation coefficient and Spearman’s Rho. The results are shown in Table 1 and Fig. 2.

**Table 1:** Summary Statistics

	Open Access (OA) ratio			APCs (USD 2018)			APCs - IF correlation		
	Diamond	Hybrid	No OA option	Range	Mean	Pearson	Significance	Spearman	Significance
Computer Science (Eng.)	2	84	12	563–3900	2276	0.0092	Weak negative	0.0036	Not sign
Computer Science (Theo.)	4	82	14	560–3900	2423	0.0841	Weak positive	0.1749	Not sign
Area studies	6	75	19	2350–3260	2917	0.2685	**Positive**	0.2788	**Significant**
Anthropology	11	71	18	1500–4200	2850	0.244	**Positive**	0.2657	**Significant**
Average	5.75	78	15.75	560–4200	2616.5	0.0571	Weak positive	0.01993	Not sign

Bold emphasizes significant positive correlations between the values

The first column in Table 1 shows the proportion of SSCI journals with different OA models: Diamond, Hybrid and No OA option. For example, in Computer Science (Engineering) 2% are Diamond OA journals, 84% are Hybrid OA journals and 12% do not offer an OA option. The OA ratio category evidences that there are more Diamond OA journals in Anthropology and Area studies than in Computer Science, but there is also a higher number of journals without OA options. In Computer Science, a vast majority of journals offer OA options, but only a few of them offer Diamond OA. By contrast, in Anthropology and Area Studies a smaller proportion of journals offer OA options, but the proportion of Diamond OA journals is higher than in Computer Science. Overall, Diamond journals are under 10%, with the exception of Anthropology. However, it should be noted that in Anthropology, more than half of the Diamond OA journals in the sample are published either in Latin-America or Spain, which means that these tend to be published in Spanish with an English abstract.^5^

The second column shows the range and the mean of APCs for each discipline. According to the results, APCs are significantly higher in Anthropology and in Area Studies. The highest mean is seen in Area Studies at 2917 USD. The range is narrower in Anthropology and in Area Studies than it is in Computer Science, which makes the latter more accessible in economic terms.

The third column shows the correlation factors between IF values and APCs. In calculating the correlation, we considered the range of IF values per discipline, which should be indifferent of absolute IF values. In this exercise, significant positive correlations between IF values and APCs were found in Area Studies and in Anthropology but not in Computer Science.

### The Relationship Between APCs and Power Purchasing Parity (PPP)

In order to demonstrate the economic disparities between countries and how OA publication costs compare in terms of Power Purchasing Power, we developed a detailed list of countries with a population of over 2 million, which we classified by world regions. Table 2 shows the country, its PPP per capita and the region it belongs to. Regions were classified according to scientometric distribution [7, 21], except for a few countries that we repositioned for reasons that we discuss below. The last column shows the ratio of the average APC divided by per capita PPP, which essentially calculates how many articles per person could be published annually in a country given equalised incomes. This is only a theoretical assessment since, in practice, individuals have multiple competing expenditures and costs for publishing articles become often secondary to more immediate living costs. Nevertheless, this simple variable is useful in showing how practically challenging OA publishing can be for researchers from different regions of the world. This list includes numerous countries in which authors could theoretically publish up to 25 or 30 OA articles per year (given their per capita PPP), but also countries where the per capita PPP per year is lower than the APCs charged for publishing even a single article under the Hybrid OA model.

**Table 2:** Article/year by per capita PPP

Country	PPP per capita	World regions	Article/year
	(2018) USD World Bank		Calculated by the av. APC (2558 USD)
Gabon	18,647	*Africa*	7.28
Botswana	17,888	*Africa*	6.99
Algeria	15,611	*Africa*	6.1
South Africa	13,774	*Africa*	5.38
Egypt	13,373	*Africa*	5.22
Tunisia	12,369	*Africa*	4.83
Namibia	11,516	*Africa*	4.5
Libya	10,797	*Africa*	4.22
Morocco	8956	*Africa*	3.5
Rep. of Congo	6881	*Africa*	2.68
Angola	6782	*Africa*	2.65
Nigeria	6030	*Africa*	2.35
Ghana	5026	*Africa*	1.96
Mauritania	4563	*Africa*	1.78
Sudan	4221	*Africa*	1.65
Côte d’Ivoire	4169	*Africa*	1.62
Zambia	4119	*Africa*	1.61
Cameroon	3820	*Africa*	1.49
Kenya	3694	*Africa*	1.44
Senegal	3675	*Africa*	1.43
Tanzania	3446	*Africa*	1.34
Lesotho	3373	*Africa*	1.38
The Gambia	2762	*Africa*	1.07
Uganda	2489	*Africa*	0.97
Chad	2427	*Africa*	0.94
Benin	2411	*Africa*	0.94
Zimbabwe	2381	*Africa*	0.93
Ethiopia	2344	*Africa*	0.91
Guinea	2277	*Africa*	0.89
Mali	2271	*Africa*	0.88
Rwanda	2231	*Africa*	0.87
Burkina Faso	1995	*Africa*	0.77
Guinea-Bissau	1951	*Africa*	0.76
Togo	1737	*Africa*	0.67
Eritrea	1657	*Africa*	0.64
Madagascar	1626	*Africa*	0.63
Sierra Leone	1618	*Africa*	0.63
South Sudan	1527	*Africa*	0.59
Liberia	1326	*Africa*	0.51
Mozambique	1294	*Africa*	0.5
Niger	1217	*Africa*	0.47
Malawi	1201	*Africa*	0.46
Dem. Rep. Congo	816	*Africa*	0.31
Burundi	733	*Africa*	0.28
Cent. Afr. Rep.	712	*Africa*	0.27
**av.**	**4972**	***Africa***	**1.94**
Singapore	98,255	*Wealthier Asia*	38.41
Hong Kong	64,794	*Wealthier Asia*	25.32
Taiwan	52,960	*Wealthier Asia*	20.7
Japan	44,549	*Wealthier Asia*	17.41
Korea	41,415	*Wealthier Asia*	16.19
**av.**	**60,395**	***Wealthier Asia***	**23.61**
Malaysia	30,815	*Less wealthy Asia*	12.04
Kazakhstan	27,494	*Less wealthy Asia*	10.74
Turkmenistan	19,526	*Less wealthy Asia*	7.63
Thailand	19,126	*Less wealthy Asia*	7.47
China	18,120	*Less wealthy Asia*	7.08
Mongolia	13,904	*Less wealthy Asia*	5.43
Sri Lanka	13,500	*Less wealthy Asia*	5.27
Indonesia	13,176	*Less wealthy Asia*	5.15
Philippines	8933	*Less wealthy Asia*	3.49
India	7795	*Less wealthy Asia*	3.04
Vietnam	7482	*Less wealthy Asia*	2.92
Uzbekistan	7337	*Less wealthy Asia*	2.86
Myanmar	6797	*Less wealthy Asia*	2.65
Bangladesh	4598	*Less wealthy Asia*	1.79
Cambodia	4323	*Less wealthy Asia*	1.68
Kyrgyz Rep.	3812	*Less wealthy Asia*	1.49
Tajikistan	3354	*Less wealthy Asia*	1.31
Nepal	2901	*Less wealthy Asia*	1.13
**av.**	**11,833**	***Less wealthy Asia***	**4.61**
		Without China*	4.48
Czech Rep.	37,423	*Non*-*Western Europe*	14.62
Slovenia	36,825	*Non*-*Western Europe*	14.39
Slovak Rep.	35,098	*Non*-*Western Europe*	13.72
Lithuania	34,829	*Non*-*Western Europe*	13.61
Estonia	33,553	*Non*-*Western Europe*	13.11
Poland	31,647	*Non*-*Western Europe*	12.37
Hungary	31,560	*Non*-*Western Europe*	12.33
Latvia	29,487	*Non*-*Western Europe*	11.52
Russia	29,032	*Non*-*Western Europe*	11.34
Croatia	26,215	*Non*-*Western Europe*	10.24
Romania	26,176	*Non*-*Western Europe*	10.23
Bulgaria	23,207	*Non*-*Western Europe*	9.07
Belarus	20,175	*Non*-*Western Europe*	7.88
Azerbaijan	17,954	*Non*-*Western Europe*	7018
Serbia	16,089	*Non*-*Western Europe*	6.28
Macedonia	15,522	*Non*-*Western Europe*	6.06
Bosnia & Herz.	13,513	*Non*-*Western Europe*	5.28
Albania	13,330	*Non*-*Western Europe*	5.21
Georgia	11,600	*Non*-*Western Europe*	4.53
Armenia	10,274	*Non*-*Western Europe*	4.01
Ukraine	9182	*Non*-*Western Europe*	3.58
Moldova	7103	*Non*-*Western Europe*	2.77
Greece	29,111	*Non*-*Western Europe*	11.38
**av.**	**23,172**	*Non*-*Western Europe*	**9.15**
Puerto Rico	39,763	*Latin America*	15.54
Chile	25,891	*Latin America*	10.12
Uruguay	23,266	*Latin America*	9.09
Mexico	20,645	*Latin America*	8.07
Argentina	20,609	*Latin America*	8.05
Dominican Rep.	18,323	*Latin America*	7.16
Costa Rica	17,644	*Latin America*	6.89
Brazil	16,111	*Latin America*	6.29
Colombia	15,021	*Latin America*	5.87
Peru	14,252	*Latin America*	5.57
Paraguay	13,471	*Latin America*	5.26
Ecuador	11,732	*Latin America*	4.58
Venezuela	10,968	*Latin America*	4.28
Dominica	9726	*Latin America*	3.8
Guatemala	8414	*Latin America*	3.28
El Salvador	8388	*Latin America*	3.27
Bolivia	7943	*Latin America*	3.1
Honduras	5817	*Latin America*	2.27
Nicaragua	5683	*Latin America*	2.22
Haiti	1875	*Latin America*	0.73
**av.**	**14.777**	***Latin America***	**5.77**
Qatar	128,487	*Middle East*	50.22
Un. Arab Em.	70,262	*Middle East*	27.46
Kuwait	66,982	*Middle East*	26.18
Saudi Arabia	55,926	*Middle East*	21.86
Oman	46,522	*Middle East*	18.18
Israel	37,855	*Middle East*	14.79
Turkey	28,270	*Middle East*	11.05
Iran	20,069	*Middle East*	7.84
Lebanon	20,027	*Middle East*	7.82
Iraq	16,926	*Middle East*	6.61
Jordan	9406	*Middle East*	3.67
Pakistan	5714	*Middle East*	2.23
Yemen	2380	*Middle East*	0.93
Afghanistan	2018	*Middle East*	0.78
**av.**	**36,489**	***Middle East***	**14.26**
		Without Israel**	14.22
		Without Turkey***	14.51
		*Without Israel and Turkey*	14.48
United States	62,517	*North America*	24.43
Canada	49,935	*North America*	19.52
**av.**	**56,226**	***North America***	**21.98**
Australia	52,363	*Oceania*	20.47
New Zealand	40,266	*Oceania*	15.74
**av.**	**46,315**	***Oceania***	**18.1**
Ireland	77,669	*Western Europe*	30.36
Norway	74,318	*Western Europe*	29.05
Switzerland	64,987	*Western Europe*	25.4
Netherlands	56,570	*Western Europe*	22.11
Iceland	54,752	*Western Europe*	21.4
Germany	52,896	*Western Europe*	20.67
Sweden	52,718	*Western Europe*	20.6
Austria	52,224	*Western Europe*	20.41
Denmark	51,840	*Western Europe*	20.26
Belgium	48,178	*Western Europe*	18.83
Finland	46,559	*Western Europe*	18.2
UK	45,642	*Western Europe*	17.84
France	45,601	*Western Europe*	17.82
Malta	44,587	*Western Europe*	17.43
Spain	40,371	*Western Europe*	15.78
Italy	39,472	*Western Europe*	15.43
Portugal	32,023	*Western Europe*	12.51
**av.**	**50,529**	***Western Europe***	**19.75**

Bold lines represent the averages of a given world region

*Typically, China is considered as part of the “developing Asia”, but, in our opinion, this categorisation makes little sense scientometrically. China is one of the most productive countries in terms of research publications, and is even a leader in several disciplines. We, thus, decided to do the calculations for the less wealthy group of countries both including and excluding China

**Israel is often considered as part of the Middle East, but in scientometrics it is often grouped with the countries of the ‘centre’ than the periphery. In terms of education, research output and research collaboration, Israel is more proximate to the US than to other countries in the Middle East. We, thus, decided to do the calculations for the Middle Eastern countries both including and excluding Israel

***Turkey is also in a special position, scientometrically speaking. It has almost equally positioned between the centre and periphery. Turkey’s historical connections (and disconnections) to Europe are notoriously diverse and complicated, thus we considered it appropriate to do the calculations for the Middle Eastern countries both including and excluding Turkey

**Fig. 3:**
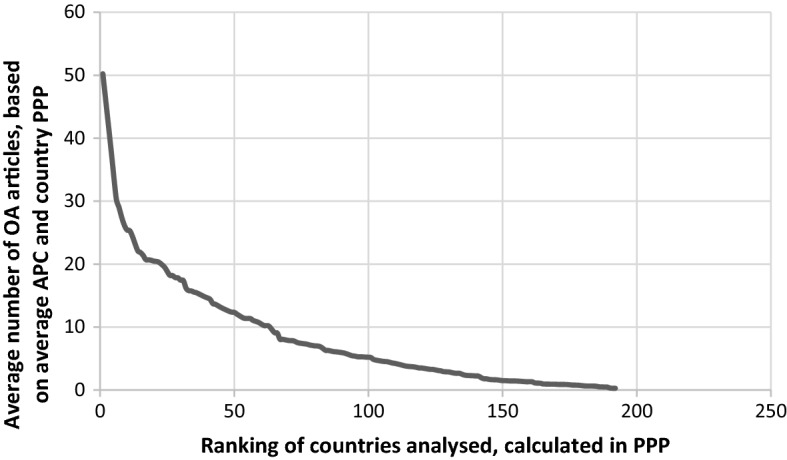
OA Articles/year for different countries

These mean and range values were also classified by world region. These did not change significantly even after repositioning the countries listed above.^6^ The two figures (Table 2 and Fig. 3) make the results more compelling. Table 2 shows how many articles could be published under an OA model in different world regions (Table 3).

**Table 3:** Average and Range of articles by world region

	Average	Range
*Wealthier Asia*	23,61	16.19–38.41
*North America*	21,98	19.52–24.43
*Western Europe*	19,75	12.51–30.36
*Australia and New Zealand*	18,10	15.74–20.47
*Middle East*	14,26	0.79–50.22
*Non*-*Western Europe*	9,15	2.77–14.63
*Latin America*	5,78	0.73–15.54
*Less Wealthy Asia*	4,63	1.13–12.04
*Africa*	1,94	0.28–7.29

Figure 3 below shows OA articles per year for different countries calculated by the average APC and PPP. The horizontal axis shows the analysed countries by World Bank ranking 2018. This ranking assigns the countries (range: 1–192) by their annual per capita GDP (PPP). For example, the first horizontal position represents Qatar, the 192th position represents Central African Republic. The horizontal axis does not show the actual PPPs, only the positions of the countries in this ranking. The vertical axis shows the number of OA articles per year that could be theoretically published by country (calculated by dividing the country per capita GDP (PPP) by the average APC). The figure shows that only a limited number of countries in the world can afford their scholars the possibility of publishing several articles per year and that the majority are found in the area of < 20 articles per year, which explains why the diagram is long-tailed. Authors in the majority of countries are unable to publish 10 or more articles per year.

### Global Distribution of Publishers

**Fig. 4:**
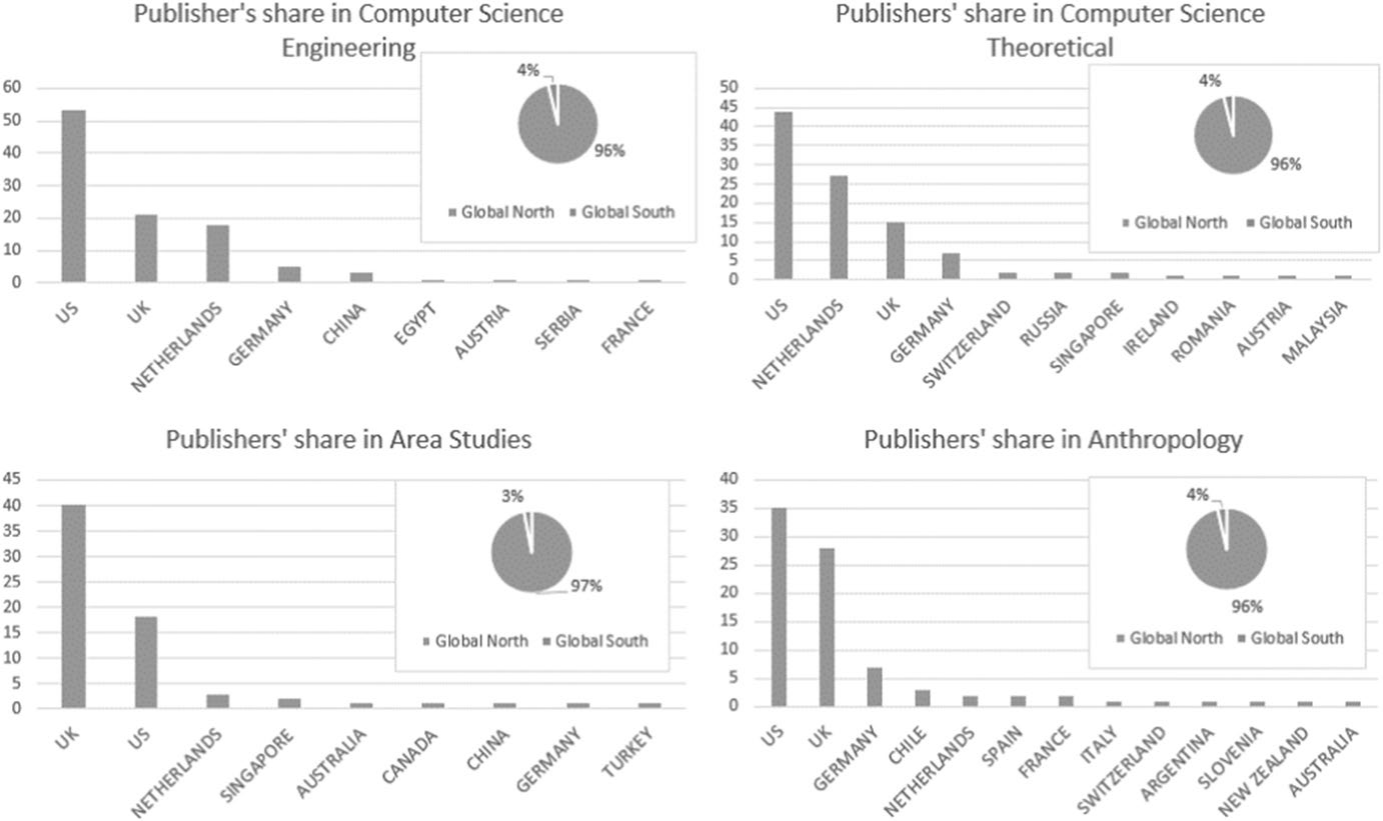
Publishers’ share in different disciplines. The columns signify the number of JCR-ranked journals in a given field of research

Our analysis of the global share of publications of different world regions shows an unbalanced picture for each of the disciplines we examined (Fig. 4). We found that the share in world publications for the Global North cumulatively is 96–97 percent. Conversely the less wealthy or peripheral regions are extremely underrepresented in terms of ‘high-impact’ published research in all the analysed fields. However, there are considerable differences within the centre and across wealthier nations. Similar to Development Studies [10], Area Studies are dominated by British journals: there are twice as many British journals as there are journals from other parts of the world combined. There is a relatively balanced US-UK dominance in the field of Anthropology with a slight US domination, with a moderate share for the Netherlands. In Computer Science (Theoretical), the share of the US is significant, followed by the share of the Netherlands and the UK. American dominance can be most explicitly seen in Computer Science (Engineering), where there are as many journals from the US as there are from all the other regions of the world combined.

## Discussion

The study found significant positive correlations between APCs and IF in Area Studies and Anthropology but not in Computer Science (Theoretical and Engineering). The average APCs in Anthropology and Area Studies were found to be considerably higher than in Computer Science (Theoretical and Engineering). Moreover, since there were more Diamond OA journals in the sample of journals in Areas Studies and Anthropology than in the sample of journals in Computer Science, the higher number of Diamond journals in the former disciplines would lower the total mean value of APCs (with a zero value being assumed for the Diamond journals). This means that the difference in APCs between Hybrid OA journals in Area studies and Anthropology and Hybrid OA journals in Computer Science is, in fact, more significant than the mean values currently suggest.

The implication of this finding is that a researcher in Area Studies or Anthropology wishing to publish under the increasingly standardised Hybrid OA model will have to pay 20–30% higher APCs than their colleagues in Computer Science. This is slightly counterintuitive since Computer Science is a resource-intensive discipline and computer scientists are anticipated to have, on average, higher (industry-based) salaries and better financing.^7^

These findings could be explained by the fact that in Computer Science there are a significant number of journals published by resourceful international associations,^8^ such as the Institute of Electrical and Electronic Engineers (IEEE) and the Association for Computing Machinery (ACM), which own a high number of journals with high IF but charge lower APCs (between 1700 and 2000 USD) relative to the standard. Since these are highly ranked journals, they moderate the effect of IF on APCs. To our knowledge, there are no such associations in Area studies and Anthropology (although exceptions do exist in subject matters such as Psychology), with most top-tier journals being in the hands of the established large publishing houses based in Northern high-income societies. Thus the average APCs are higher in these disciplines, and the moderating effect of resourceful associations is relatively absent.

Both Anthropology and Area Studies (both with relatively higher APCs than Computer Science) were found to be dominated by Northern publishers, which suggests a relationship between western dominance in publishing and the level of APCs charged by journals. We thus believe that the relationship IF-APCs in these disciplines is largely mediated by the geographic location of the publishers. Many of the established publishers (Oxford Academic, Cambridge Core, Sage, Springer, Taylor and Francis) charge very high fixed APCs (typically around 3000 USD - Elsevier might be noted as an exception, with APCs varying from 1400 USD to 3800 USD or higher). Since such established publishers tend to own journals with higher IF values, the positive connection between high IF and APCs in these subject matters could be reinforced by their dominance in the publications landscape.

Where Anthropology is concerned, this has been a highly exclusionary and oligopolistic publishing market. Since competition in this field has been relatively low (as in the form of professional associations or alternative publishing options seen in Computer Science), prices have tended to be higher. It is not unlikely that these have been determined by the few prominent publishers in the field in line with the principles of oligopolistic market behaviour. And since Anthropology is Global North-dominated and Computer Science is more internationalised, this would suggest a correlation between high IF or APCs and inequalities in the geographic distribution of knowledge production. In Anthropology, most publishers (excepting Latin American publishers) are primarily located in the Global North and can afford to expand their ownership of high-impact journals, reflecting the norms of competition in this region. More internationalised disciplines, like Computer Science, which is anticipated to display a more international distribution of publishers, would be influenced by other parameters reflecting different publishing environments. In other words, the examined subject areas are dominated by more or less internationally distributed publishers, which shapes their interest in IF journals, the kind of market competition they face, and subsequently the APCs they choose/are able to charge.

The analysis conducted pointed to relatively high APCs being charged by the journals in the subject areas examined, but especially in less professionalised disciplines (Area Studies and Anthropology). In parallel, the calculations of the theoretical OA papers/Year that could be published per country demonstrated that OA publishing (Gold or Hybrid OA) is theoretically infeasible in most parts of the world due to financial constraints. The overall implication seems to be that authors in Southern regions of the world will be challenged to publish as prolifically as their Northern peers in *pareto* optimal conditions, but will be especially challenged to publish in Global North-dominated subject areas and journals, such as in Anthropology and Area Studies. In sum, in subject areas that are dominated by Northern publishers, the level of APCs charged and IF will move together, which combined with the existing economic inequalities among countries, are anticipated to grow the disparities between Northern and Southern researchers.

However, it is positive to see the relatively higher number of Iberoamerican (Spanish, Chilean, and Argentinean) journals that publish under a Diamond OA model, in contrast to most Anglophone publishers. By insisting to publish in Spanish (by which we mean the full text, not merely the abstracts as Anglophone publishers increasingly do), Iberoamerican publishers offer a different publishing model that can, in fact, subvert the current world order in publishing. This example suggests that linguistic diversity and a decentralisation of publishing from the Global North to the Global South through a wider diffusion of publishing houses and platforms in the world constitutes a positive direction forward.

## Limitations

In the current study, we included all the journals we could identify on the WoS that charge APCs under the subject areas we considered. We did not limit ourselves to OA journals that would meet the preliminary criteria of Plan S standards, for example, by looking at the DOAJ list. Still, the study can inform Plan S debates because it evidences clear patterns in APCs and how these could be affected through the shift to a Gold OA or Hybrid OA publishing model

It should be stressed that the calculations in this article concern correlations and do not claim to establish a causal relationship. To demonstrate that a higher IF drives APCs or higher APCs contribute to higher IFs it would be necessary to conduct regression analyses with different models using continuous variables that could predict causation. The causal direction, however, is less important to the argument of this paper: IFs and APCs seem to move together in disciplines that are dominated by Global North publications and publishers, which is likely to perpetuate existing asymmetries.

## Footnotes

1 As in Istratii and Demeter [16], we define the Global South/North division solely on the basis of scientometric indicators in the humanities and social sciences [7, 8]. ‘Global North’ incorporates the US, Canada, Australia and New Zealand, the UK, Western Europe, Israel and the Asian countries like Japan, Hong Kong, Singapore, South Korea and Taiwan. ‘Global South’ includes Latin America (including South and Central America), Eastern Europe, Asia (except those societies mentioned), the Middle East and Africa (and parts of Oceania except Australia and New Zealand).

2 This is a strictly technical definition of impact. In our experience, this is a very problematic conceptualisation of impact because it disregards completely how accessible and impactful the research is on communities and in informing societal issues.

3 Articles published in what are considered to be ‘predatory’ journals (a designation we find problematic since it seems to suggest that typical profit-oriented journals are not predatory), typically remain invisible and uncited, and, consequently, these journals are not listed on the WoS and lack IF metrics.

4 It should be clarified here that by ‘Global South’ we are not limited to those countries that many journals list as ‘developing’ countries (a term we find derogatory and which we hope publishers will cease perpetuating) and to which they offer the option of reduced fees or a full fee waivers.

5 The journals in question are Chungara-Revista de Antropologia Chilena, Estudios Atacamenos, Trabajos de Prehistoria, AIBR-Revista de Antropologia Iberoamericana, Intersecciones en Antropología and Magellania. It may be observed that Latin American publishing in Spanish represents one of the few counterforces to the prevalent Anglophone publishing landscape, but this is in part because Spanish is widely spoken in the world. Contrary to this, while Chinese capital has increasingly penetrated Anglophone publishing houses, Chinese publishers have not tried to subvert the dominance of Anglophone epistemology in the way in which Latin American journals have done [1].

6 It should be mentioned here that we did not weigh countries but considered them as equals when calculating world region ratios.

7 It is not possible to provide a global data-based analysis on salaries (to our knowledge there is no database listing salaries in different disciplines and in different world regions), however, it has been convincingly demonstrated that geopolitical locality and disciplinary background significantly affect funding and salaries (see e.g. Rumbley et al. [23]).

8 Associations that have journals which are (1) published by their own (and not by giant publishing houses) and (2) indexed in WoS SSCI are not typical in other disciplines. For example, we have ICA and NCA in communications studies, both are international associations and have flagship journals. But all ICA and NCA journals are published by giant publishers as Taylor and Francis, Oxford University Pres or SAGE, thus they charge the same APC as other journals from these publishers. As opposed to this, IEEE and ACM are associations on the one hand, and, on the other hand, they also publish their journals on their own (and not through giant publishers). That’s why they can use a lower APC rate than those journals that are published by giant international publishing houses.

## References

[1] Asheulova N, Dushina S, Prpić K, van der Weijden I, Asheulova N (2014). Research career development in Russia: the role of international mobility. (Re)searching Scientific Careers.

[2] Bennett K, PloAlastrué R, Pérez-Llantada C (2015). Towards an epistemological monoculture: Mechanisms of epistemicide in European research publication. English as a Scientific and Research Language.

[3] Canagarajah SA (2002). A Geopolitics of academic writing.

[4] Cummings S, Hoebink P (2016). Representation of academics from developing countries as authors and editorial board members in scientific journals: does this matter to the field of development studies?. Eur. J. Develop Res..

[5] Curry MJ, Lillis T (2018). Global academic publishing. Policies, perspectives and pedagogies.

[6] De Castro P, Salinetti S (2004). Quality of grey literature in the open access era: privilege and responsibility. Pub Res Q..

[7] Demeter M (2018). Changing center and stagnant periphery in communication and media studies: national diversity of major international journals in the field of communication from 2013 to 2017. Int J Commun..

[8] Demeter M (2018). The winner takes it all: international inequality in communication and media studies today. J Mass Commun Q..

[9] Demeter M (2019). Open access movements: emancipation or hypocrisy?. KOME: An International Journal of Pure Communication Inquiry.

[10] Demeter M (2020). Academic knowledge production and the Global South. Questioning inequality and underrepresentation.

[11] Efranmanesh M, Tahira M, Abrizah A (2016). The publication success of 102 nations in Scopus and the performance of their Scopus-indexed journals. Publishing Research Quarterly..

[12] Ejikeme AN, Ezema IJ (2019). The potentials of open access initiative and the development of institutional repositories in Nigeria: implications for scholarly communication. Pub Res Q..

[13] Escobar A (1995). Encountering development: the making and unmaking of the third world.

[14] Heilbron J, Sorá G, Boncourt T (2018). The social and human sciences in global power relations.

[15] Istratii R. Substantiating ‘development’: toward an epistemology-sensitive development as freedom? SOAS African Development Forum (ADF), 2018. Accessed from https://eprints.soas.ac.uk/30606/.

[16] Istratii R, Demeter M. Plan S and the ‘opening up’ of scientific knowledge: a critical commentary. Decolonial Subversions. 2020; 13–21.

[17] Istratii R, Lewis, A. Applying a decolonial lens to research structures, norms and practices in higher education institutions: conversation event report. In: Applying a Decolonial Lens to Research Structures, Norms and Practices in Higher Education Institution. 2019. Retrieved from https://eprints.soas.ac.uk/32053.

[18] Kapoor I (2004). Hyper-self-reflexive development? Spivak on representing the third world ‘other’. Third World Quarterly..

[19] Koutras N (2015). The open access in the context of the globalizing world. Pub Res Q..

[20] Koutras N (2020). Open access publishing in the European Union: the example of scientific works. Pub Res Q..

[21] Lauf E (2005). National diversity of major international journals in the field of communication. Journal of Communication..

[22] Open Letter in Support of Funder Open Publishing Mandates. *Michael Eisen*. http://michaeleisen.org/petition/index.php. Accessed 7 May 2019.

[23] Rumbley LE, Pacheco IF, Altbach PG (2008). International comparison of academic salaries. An exploratory study.

[24] Saurin TA (2016). Ethics in publishing: complexity science and human factors offer insights to develop a just culture. Sci Eng Ethics.

[25] Schott T (1998). Ties between center and periphery in the scientific world-system: accumulation of rewards, dominance and self-reliance in the center. J World-Syst Res.

[26] Shenhav YA (1986). Dependency and compliance in academic research infrastructures. Sociological Perspectives..

[27] Siversten G (2016). Patterns of internationalization and criteria for research assessment in the social sciences and humanities. Scientometrics.

[28] Statement by European Commission: ‘Plan S’ and ‘cOAlition S’ – Accelerating the transition to full and immediate Open Access to scientific publications. *European Commission*, 4 September 2018, https://ec.europa.eu/commission/commissioners/2014-2019/moedas/announcements/plan-s-and-coalition-s-accelerating-transition-full-and-immediate-open-access-scientific_en. Accessed 17 May 2015.

[29] Van Noorden R. Researchers sign petition backing plans to end paywalls. Nature News. *2018; 4*. Retrieved from https://www.nature.com/articles/d41586-018-07632-2?utm_source=briefing. Accessed 7 May 2019.

[30] Xia JA, Smith MP (2018). Alternative journal impact factors in open access publishing. Learn Publ..

[31] Xia JA (2019). Preliminary study of alternative open access journal indexes. Pub Res Q.

